# Joint modeling of genetically correlated diseases and functional annotations increases accuracy of polygenic risk prediction

**DOI:** 10.1371/journal.pgen.1006836

**Published:** 2017-06-09

**Authors:** Yiming Hu, Qiongshi Lu, Wei Liu, Yuhua Zhang, Mo Li, Hongyu Zhao

**Affiliations:** 1 Department of Biostatistics, Yale School of Public Health, New Haven, Connecticut, United States of America; 2 Peking University, Beijing, China; 3 Shanghai Jiao Tong University, Shanghai, China; 4 Program of Computational Biology and Bioinformatics, Yale University, New Haven, Connecticut, United States of America; 5 Department of Genetics, Yale University School of Medicine, New Haven, Connecticut, United States of America; 6 Clinical Epidemiology Research Center (CERC), Veterans Affairs (VA) Cooperative Studies Program, VA Connecticut Healthcare System, West Haven, Connecticut, United States of America; Case Western Reserve University, UNITED STATES

## Abstract

Accurate prediction of disease risk based on genetic factors is an important goal in human genetics research and precision medicine. Advanced prediction models will lead to more effective disease prevention and treatment strategies. Despite the identification of thousands of disease-associated genetic variants through genome-wide association studies (GWAS) in the past decade, accuracy of genetic risk prediction remains moderate for most diseases, which is largely due to the challenges in both identifying all the functionally relevant variants and accurately estimating their effect sizes. In this work, we introduce PleioPred, a principled framework that leverages pleiotropy and functional annotations in genetic risk prediction for complex diseases. PleioPred uses GWAS summary statistics as its input, and jointly models multiple genetically correlated diseases and a variety of external information including linkage disequilibrium and diverse functional annotations to increase the accuracy of risk prediction. Through comprehensive simulations and real data analyses on Crohn’s disease, celiac disease and type-II diabetes, we demonstrate that our approach can substantially increase the accuracy of polygenic risk prediction and risk population stratification, i.e. PleioPred can significantly better separate type-II diabetes patients with early and late onset ages, illustrating its potential clinical application. Furthermore, we show that the increment in prediction accuracy is significantly correlated with the genetic correlation between the predicted and jointly modeled diseases.

## Author summary

Genetic risk prediction plays a significant role in precision medicine. Accurate prediction models could have great impact on disease prevention and treatment strategies. However, prediction accuracies for most complex diseases remain moderate mainly due to the challenges in identifying and quantifying the effects of genetic variants from millions of markers, limited access to individual-level genotype data, and lack of efficient computational methods. Up to now, most methods have been focused on predicting disease risk using data from a single trait. With the discovery of genetic correlations among many complex diseases, incorporating data of genetically correlated diseases could have the potential to increase prediction accuracy. Current statistical methods are not able to fully exploit the richness of these kinds of data to take into account the shared genetic architecture. To make use of commonly available GWAS summary statistics, we propose a novel method to address these challenges by jointly modeling genetically correlated diseases and integrating genomic functional annotations. We demonstrate the substantial improvement in accuracy in both extensive simulation studies and real data analysis of Crohn’s disease, celiac disease and type-II diabetes. Furthermore, we show that the increment in prediction accuracy is significantly correlated with the genetic correlation between the predicted and jointly modeled diseases.

## Introduction

Achieving accurate disease risk prediction using genetic information is a major goal in human genetics research and precision medicine. Accurate prediction models will have great impacts on disease prevention and treatment strategies[1]. Various approaches that utilize genome-wide data in genetic risk prediction have been proposed, including machine-learning models trained on individual-level genotype and phenotype data[2–7], and polygenic risk scores (PRS) derived from genome-wide association study (GWAS) summary statistics [8, 9]. Despite the potential information loss in summary data, PRS-based approaches have been widely adopted in practice due to computational efficiency and the easy accessibility of GWAS summary level data[10, 11]. However, prediction accuracies for most complex diseases remain moderate, which is largely due to the challenges in both identifying all the functionally relevant variants and accurately estimating their effect sizes in the presence of linkage disequilibrium (LD) [12].

Integrating external information, e.g. pleiotropy [2, 3], LD [9], and functional annotations[13] has been shown to effectively address these challenges. Maier et al.[3] and Li et al.[2] showed that joint modeling of correlated traits could increase the prediction accuracy using individual level genotype data for psychiatric disorders and autoimmune diseases. Using summary level data, Hu et al.[13] proposed a single-trait risk prediction framework explicitly modeling LD and functional annotations, which consistently improves prediction accuracy for complex diseases. Furthermore, integrative genomic functional annotation, coupled with the rich collection of summary statistics from GWAS, have enabled increased statistical power in several different settings [14, 15]. Here, we introduce PleioPred (available at https://github.com/yiminghu/PleioPred), a principled framework that integrates GWAS summary statistics of genetically correlated diseases with various types of annotation data and reference genotype panels to improve risk prediction accuracy. Incorporating data from related traits and functional annotations increases the effective sample size and statistical power to detect functionally relevant variants, especially when diseases share similar genetic architecture. We compare PleioPred with state-of-the-art single-trait PRS-based approaches and demonstrate its consistent improvement in risk prediction performance using real data of multiple complex diseases.

We first apply PleioPred to Crohn’s disease (CD), celiac disease (CEL) and type-II diabetes (T2D) by jointly modeling them with known correlated diseases (i.e. CD with Ulcerative Colitis (UC); CEL with UC; T2D with coronary artery disease (CAD)) and show a statistically significant improvement in prediction performance in independent validation cohort over single-trait models. By comparing two-trait prediction model with and without functional annotations in both simulation and real data analysis, we demonstrate that functional annotation may further improve the performance of joint modeling. Furthermore, we show that PRS calculated from PleioPred can effectively partition T2D patients by their age of onset, indicating the potential clinical usage of our approach[16, 17]. Through jointly modeling T2D with a wide spectrum of diseases, we demonstrate that the increment in prediction accuracy is significantly correlated with the genetic correlations between T2D and the jointly modeled diseases.

## Results

### Methods overview

We propose a Bayesian framework to incorporate functional annotations and pleiotropy. We assume throughout the report that the phenotypes of two diseases $Y_{N_{1}\times 1}^{\left(1\right)},\;Y_{N_{2}\times 1}^{\left(2\right)}$ and the genotypes $X_{N_{1}\times M},\;Z_{N_{2}\times M}$ are standardized with mean zero and variance one. When phenotypes are binary, $Y_{N_{1}\times 1}^{\left(1\right)}$ and $Y_{N_{2}\times 1}^{\left(2\right)}$ denote disease liabilities instead [18, 19]. Here N_1_ and N_2_ denote the sample sizes for the two diseases and M is the number of markers. We assume a linear model with genotype matrices, effect sizes (*β* and *γ*) and random errors (*ε* and *δ*) mutually independent as follows

$$Y_{N_{1}\times 1}^{\left(1\right)}=X_{N_{1}\times M}\beta_{M\times 1}+\varepsilon_{N_{1}\times 1}$$

$$Y_{N_{2}\times 1}^{\left(2\right)}=Z_{N_{2}\times M}\gamma_{M\times 1}+\delta_{N_{2}\times 1}$$

We also assume that the effect sizes of different SNPs are independent. As for random errors, we assume that

$$\left(\begin{array}{c} \varepsilon \\ \delta \end{array}\right)\sim N\left(\left(\begin{array}{c} 0\\ 0 \end{array}\right),\left[\begin{array}{cc} \left(1-h_{1}^{2}\right)I_{N_{1}} & S\\ S^{T} & \left(1-h_{2}^{2}\right)I_{N_{2}} \end{array}\right]\right)$$

$$S_{ij}=\{\begin{array}{ll} \rho_{e} & \text{if individual i in study }1\text{ and individual j in study }2\text{ are the same}\\ 0 & \text{if individual i in study }1\text{ and individual j in study }2\text{ are different} \end{array}$$

where $h_{1}^{2}$ and $h_{2}^{2}$ denote the heritability of two diseases and *ρ*_*e*_ measures the covariance within the overlapping individuals between two studies. Denote the LD matrix and marginal effect size estimator from GWAS as: ${\hat{D}}_{1}=\frac{1}{N_{1}}X^{T}X,\;\;{\hat{D}}_{2}=\frac{1}{N_{2}}Z^{T}Z,\;\;\tilde{\beta }=\frac{1}{N_{1}}X^{T}Y^{\left(1\right)}$ and $\tilde{\gamma }=\frac{1}{N_{2}}Z^{T}Y^{\left(2\right)}$. In practice, ${\hat{D}}_{1}$ and ${\hat{D}}_{2}$ can be estimated from a reference panel and we therefore denote the LD matrix as $\hat{D}$ for convenience. Then following the derivation in Hu et al. [13], we can derive the conditional distribution of GWAS summary statistics as

$$\begin{array}{c} \tilde{\beta }\\ \tilde{\gamma } \end{array}|\begin{array}{c} \beta \\ \gamma \end{array},\hat{D}\sim N\left(\left(\begin{array}{c} \hat{D}\beta \\ \hat{D}\gamma \end{array}\right),\;\left[\begin{array}{cc} \frac{1-h_{1}^{2}}{N_{1}}\hat{D} & \frac{N_{S}\rho_{e}}{N_{1}N_{2}}\hat{D}\\ \frac{N_{S}\rho_{e}}{N_{1}N_{2}}\hat{D} & \frac{1-h_{2}^{2}}{N_{2}}\hat{D} \end{array}\right]\right)$$

where *N*_*s*_ is the number of overlapping samples between the two studies. When *N*_*s*_ is relatively small, we can discard terms with $\frac{N_{S}\rho_{e}}{N_{1}N_{2}}$ to reduce the computation burden.

We first consider an infinitesimal model to account for a polygenic genetic architecture. We assume that the effect sizes follow a multivariate normal distribution:

$$\left(\begin{array}{c} \beta \\ \gamma \end{array}\right)\sim N\left(\left(\begin{array}{c} 0\\ 0 \end{array}\right),\left[\begin{array}{cc} diag\left(\sigma_{1i}^{2}\right) & \rho_{g}*diag\left(\sigma_{1i}\sigma_{2i}\right)\\ \rho_{g}*diag\left(\sigma_{1i}\sigma_{2i}\right) & diag\left(\sigma_{2i}^{2}\right) \end{array}\right]\right)$$

where $\sigma_{1i}^{2}$ and $\sigma_{2i}^{2}$ denote the variance of effect sizes of SNP *i* and *ρ*_*g*_: = *cor*(*β*_*i*_, *γ*_*i*_), represents the genetic correlation between two diseases. This is equivalent to a multivariate random effects model with various variance components. Suppose that the whole genome is partitioned into *K* functional regions *A*_1_, …, *A*_*K*_. We assume that the effect size of a SNP depends on the functional regions it falls in and the effect sizes are additive in the overlapping regions. To be specific, we have

$$var\left(\beta_{i}\right)=\sigma_{1i}^{2}=\;\sum_{c:i\in A_{c}}\tau_{1c}$$

$$var\left(\gamma_{i}\right)=\sigma_{2i}^{2}=\;\sum_{c:i\in A_{c}}\tau_{2c}$$

where *τ*_*jc*_ denotes the variance of the effect size of SNPs on disease *j* falling in *A*_*c*_ alone. In the random effects model, the variance of effect size can be interpreted as heritability and thus for convenience, we will use heritability of SNP *i* instead of the variance of effect size in the rest of the manuscript.

Details on parameter estimation are described in **Methods**. When all the parameters are specified, we can estimate the expectation of the effect sizes given the marginal effect size estimators of two diseases. The PRSs are defined as

$$PRS_{1}=\sum_{j=1}^{M}X_{j}E(\beta_{j}|\tilde{\beta },\tilde{\gamma },\hat{D})$$

$$PRS_{2}=\overset{M}{\underset{j=1}{\sum }}Z_{j}E(\gamma_{j}|\tilde{\beta },\tilde{\gamma },\hat{D})$$

Finally, we treat *ρ*_*g*_ as a tuning parameter and the posterior expectation of the effect sizes can be calculated in closed form (Methods).

In practice[9, 13], we note that a sparse model yields higher accuracy for most diseases. Moreover, the infinitesimal model assumption is relatively strong in some cases. For example, two related diseases may only share some causal variants and have no correlation among the effect sizes or the correlation structures may vary across the genome. We therefore propose a hierarchical Bayesian model with a more general assumption and we refer to this framework as the non-infinitesimal model. Under this model, we assume that the effect sizes follow a mixture distribution.

$$\left(\begin{array}{c} \beta_{i}\\ \gamma_{i} \end{array}\right)|\vec{p}≜\left(p_{11},p_{10},p_{01},p_{00}\right)\sim p_{11}\left(\begin{array}{c} N\left(0,\frac{\sigma_{1i}^{2}}{p_{11}+p_{10}}\right)\\ N\left(0,\frac{\sigma_{2i}^{2}}{p_{11}+p_{01}}\right) \end{array}\right)+p_{10}\left(\begin{array}{c} N\left(0,\frac{\sigma_{1i}^{2}}{p_{11}+p_{10}}\right)\\ \delta_{0} \end{array}\right)+p_{01}\left(\begin{array}{c} \delta_{0}\\ N\left(0,\frac{\sigma_{2i}^{2}}{p_{11}+p_{01}}\right) \end{array}\right)+p_{00}\left(\begin{array}{c} \delta_{0}\\ \delta_{0} \end{array}\right)$$

$$\vec{p}\sim Dirichlet\left(\alpha \right)$$

That is, the effect sizes of SNP *i* for the two diseases follow a mixture distribution with two independent normal distribution (when SNP *i* is causal in both diseases), joint normal and point mass (when SNP *i* is causal in only one diseases) and joint point mass (when SNP *i* is not causal in either disease) [20]. Although we do not have closed form solution for the posterior expectation of the effect sizes, we use Markov Chain Monte Carlo (MCMC) to sample from the posterior distribution of the effect sizes to estimate the posterior expectation (Methods).

For both infinitesimal and non-infinitesimal models, we used a total of 61 different annotation categories, including functional genome predicted by GenoCanyon scores [14], GenoSkyline tissue-specific functionality scores of 7 tissue types [15], and 53 baseline annotations for diverse genomic features [21]. More specifically, GenoCanyon is a statistical framework to predict functional regions in the human genome through integrative analysis of ENCODE epigenomic data and multiple conservation metrics [14]. Later we further extended the framework and developed GenoSkyline, which aimed to predict tissue-specific functionality [15]. We smoothed GenoCanyon scores by a 10Kb window, a strategy previously shown to improve robustness of functionality prediction [22]. The smoothed GenoCanyon annotation and raw GenoSkyline annotations of seven tissue types were dichotomized based on a cutoff of 0.5. The regions with GenoCanyon or GenoSkyline scores greater than the cutoff are interpreted as non-tissue-specific or tissue-specific functional regions in the human genome. Such dichotomization has been previously shown to be robust against the cutoff choice [15].

We compare the prediction performance of eight methods, corresponding to infinitesimal and non-infinitesimal versions of single-trait and two-trait approaches with and without functional annotations. As shown in [9, 13], LDpred and AnnoPred outperform other state-of-the-art PRS methods, we therefore use these two approaches as the representative single-trait prediction methods.

- AnnoPred-inf/AnnoPred: single-trait prediction model with 61 functional annotations
- LDpred-inf/LDpred: single-trait prediction model without functional annotations, corresponding to a special case of AnnoPred when assuming only one annotation covering the whole genome
- PleioPred-anno-inf/PleioPred-anno: two-trait prediction model with 61 functional annotations
- PleioPred-inf/PleioPred: two-trait prediction model without functional annotations, corresponding to a special case of PleioPred-anno when assuming only one annotation covering the whole genome

All of these methods studied require a pre-specified tuning parameter except for PleioPred and PleioPred-anno. To select a suitable tuning parameter, we divided the independent testing dataset (individual level genotype and phenotype data) into two equal parts (A and B, non-overlapping), and selected the tuning parameters by optimizing prediction accuracy on dataset A. We then evaluated prediction accuracy using the remaining half of testing data, i.e. dataset B. Finally, we repeated the analysis one more time by choosing the tuning parameter on dataset B while evaluating the prediction accuracy on dataset A. Results from these two separate analyses were averaged to quantify model performance. Ideally, the parameter should be tuned in an independent cohort and then evaluated in another independent cohort. However, it is very challenging to find two independent cohorts without any overlapping samples with the training GWAS and we therefore chose a cross-validation scheme. In real data analysis, tuning the parameter within the same cohort may lead to a little bit over-optimistic results due to possible shared confounders. However, the proposed non-infinitesimal models address this issue via a hierarchical Bayesian approach to avoid tuning parameter and thus result in more robust and generalizable estimation. Besides the methods discussed above, we have also compared the performance of proposed joint models with a recently developed multi-trait analysis tool (MTAG [23]). Following the Polygenic Prediction section in their bioRxiv preprint (page 8), we first applied MTAG to GWAS summary statistics to get the multi-trait adjusted p values and effect sizes and then used the generated summary statistics as input to LDpred. The AUC of LDpred with MTAG adjusted summary statistics and all other four methods are shown in S7 Table. Our method outperformed all other methods including MTAG. Notably, MTAG outperformed LDpred in Crohn’s disease but its performance was even slightly worse than LDpred for celiac disease and type-II diabetes.

### Simulations

We first performed simulations to demonstrate PleioPred’s ability to improve risk prediction accuracy. We simulated traits from GERA (dbGaP access number phs000674.v1.p1) genotype data, which contains 61,172 individuals genotyped for 670,176 SNPs. More specifically, we randomly selected ~28,000 individuals as training set to calculate the summary statistics for disease 1 and another ~28,000 for disease 2. The remaining ~5000 individuals were used for testing. Throughout the simulation we used genotype data of chromosome 1 (50,279 SNPs) to generate phenotypes. We first generated two annotations and each annotation was simulated by randomly selecting 10% of the genome, denoted as *A*_1_ and *A*_2_. Denote the heritability of each trait as $h_{1}^{2}$ and $h_{2}^{2}$ (both 30%) and the number of causal variants as *m*_1_ and *m*_2_ (both 300). Causal variants were generated as follows: one third of causal variants were selected from *A*_1_, one third from *A*_2_ and the rest from (*A*_1_⋃*A*_2_)^*C*^, of which *p* of the causal variants was shared by both diseases (0.2 and 0.8). Effect sizes of causal variants were sampled from $N\left(0,\frac{h_{1}^{2}}{m_{1}}\right)$ and $N\left(0,\frac{h_{2}^{2}}{m_{2}}\right)$. We also randomly selected 5000 individuals and 10000 individuals from the training data of disease 1 and 2 respectively to calculate summary statistics in order to study the effect of unbalanced sample sizes on the increment of prediction accuracy.

**Fig 1:**
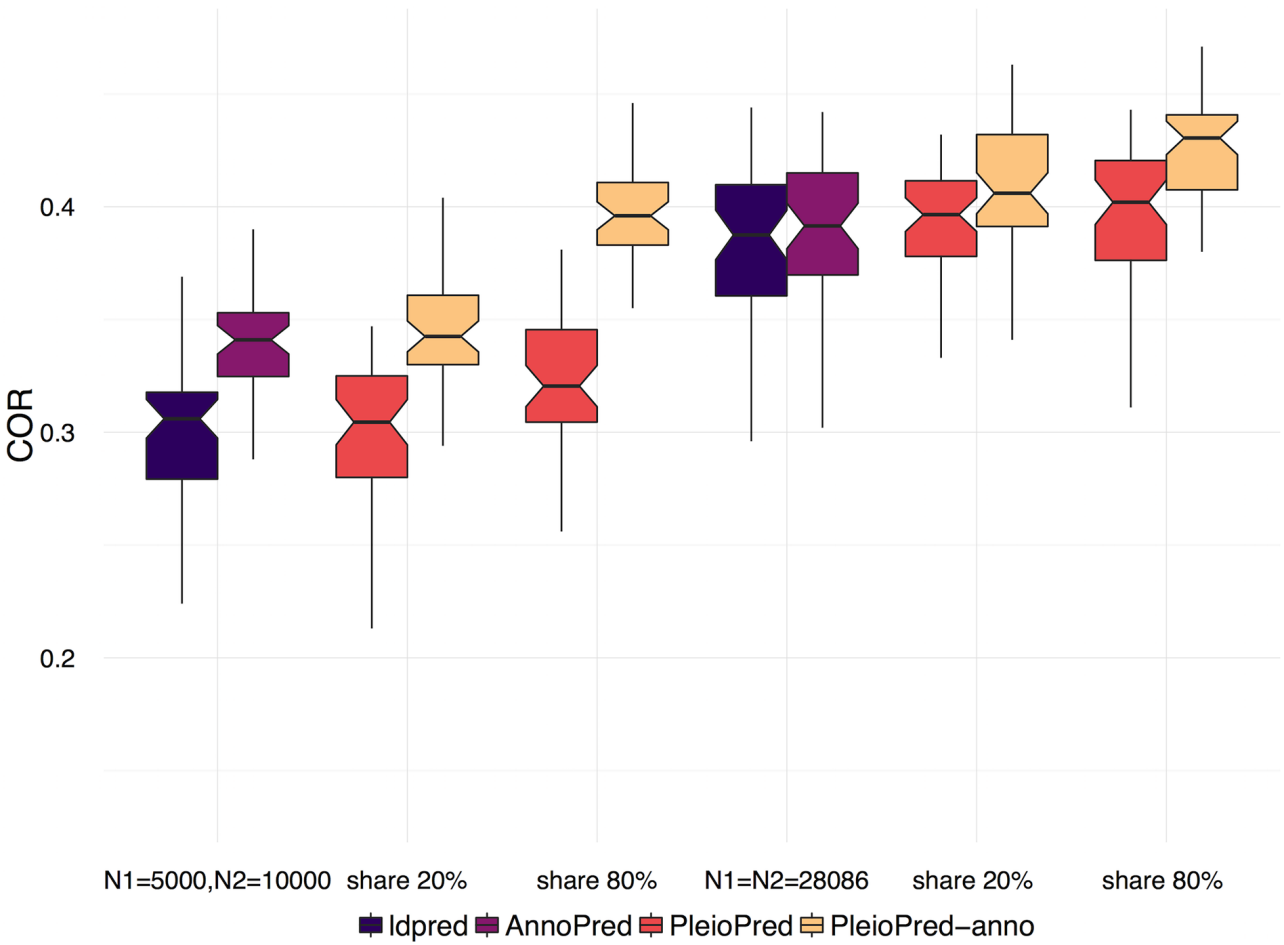
Prediction accuracy of non-infinitesimal models in simulated data. We trained the models with equal training sample sizes (*N*_1_ = *N*_2_ = 28068, right panel) and unequal training sizes (*N*_1_ = 5000, *N*_2_ = 10000, left panel). Prediction accuracy was measured by correlation between simulated traits and predicted PRS.

Correlations between simulated and predicted traits of disease 1 were calculated from 50 replicates under different simulation settings. PleioPred-anno showed the best prediction performance in all settings (Fig 1). The performance of the two-trait model improves as the proportion of shared causal variants increases. In the unbalanced case when the sample size of disease 1 is smaller than that of disease 2, we observed a larger increment in prediction accuracy, indicating that the benefit of integrating large GWAS of genetically correlated diseases and functional annotations when the sample size of disease of interest is moderate.

### Real data analysis

To further illustrate the improvement in risk prediction accuracy, we first applied PleioPred to Crohn’s disease (CD), celiac disease (CEL) and type-II diabetes (T2D). We jointly modeled CD with ulcerative colitis (UC), CEL with UC, and T2D with coronary artery disease (CAD). We trained PleioPred using publicly accessible GWAS summary statistics and evaluated risk prediction performance using individual-level genotype and phenotype data from cohorts independent from the training GWAS samples. The training summary statistics for the two autoimmune disease include the training summary statistics are from the International Inflammatory Bowel Disease Genetics Consortium (IIBDGC; CD: N_case_ = 6,333 and N_control_ = 15,056, with samples from the Wellcome Trust Case Control Consortium (WTCCC) removed from the meta-analysis), a CEL GWAS with 4,533 cases and 10,750 controls [24], a UC GWAS from IIBDGC (N_case_ = 6,687 and N_control_ = 19,718). For the validation data, we merged the CD cases from WTCCC (N_case_ = 1,829) and CEL cases from the National Institute of Diabetes and Digestive and Kidney Diseases study (NIDDK, N_case_ = 1,716) with healthy controls from the Resource for Genetic Epidemiology Research on Aging Cohort (GERA, N_control_ = 5,488). For T2D, we trained the model on summary data from the Diabetes Genetics Replication and Meta-analysis study (DIAGRAM, N_case_ = 12,171 and N_control_ = 56,862) [25] and the Coronary ARtery DIsease Genome wide Replication and Meta-analysis study (CARDIoGRAM, N_case_ = 22,233 and N_control_ = 64,762)[26]. Samples from the Northwestern NUgene Project (N_case_ = 662 and N_control_ = 517) [27] were used for validation. Details for each training GWAS summary statistics and independent testing cohorts are provided in S1 Text and S3 and S4 Tables.

**Fig 2:**
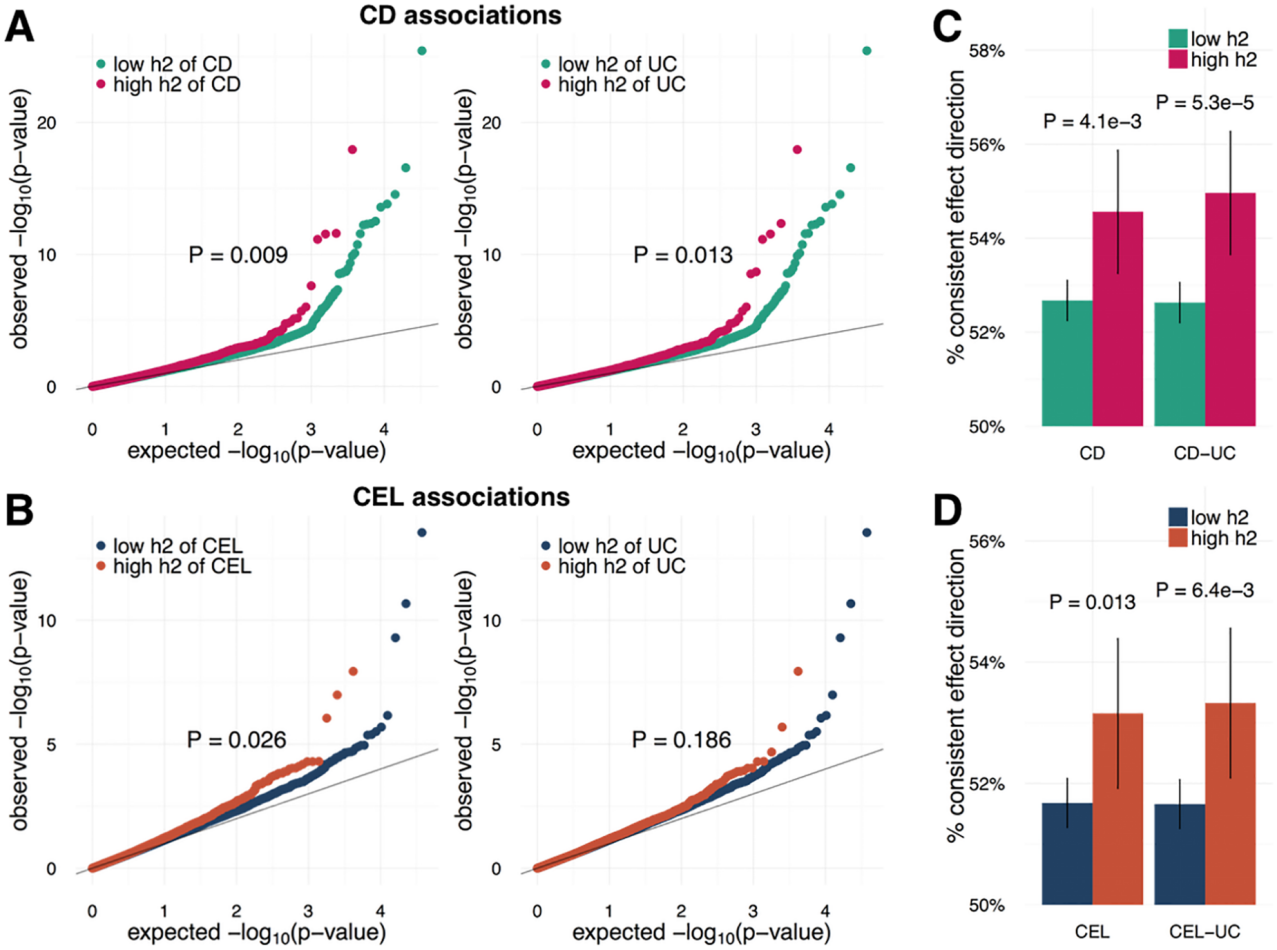
Evaluating effectiveness of annotations and per-SNP heritability. **(A, B)** Comparing signal strengths of SNPs with high and low heritability of related diseases in independent validation cohorts. Both SNPs with higher heritability of testing disease and related disease have significantly stronger associations across two independent and well-powered testing datasets (N>3,000, **(A)** Crohn’s disease; **(B)** Celiac disease.). P-values were calculated using one-sided Kolmogorov-Smirnov test. **(C, D)** Comparing consistency of SNPs’ effect direction between training and testing datasets. Each bar quantifies the proportion of SNPs with consistent effect directions. P-values were calculated using one-sided two-sample binomial test. **(C)** Crohn’s disease; **(D)** Celiac disease.

We evaluated the effectiveness of the per-SNP heritability estimated from functional annotations of the two autoimmune diseases (i.e. CD, CEL) with well-powered testing cohorts (N>3,000). Interestingly, not only the per-SNP heritability of the testing diseases (CD and CEL) but those of related diseases (UC) could effectively identify SNPs with large effect sizes (Fig 2A and 2B) and consistent effect directions in independent validation cohorts (Fig 2C and 2D), which shows that functional annotations can effectively prioritize shared causal variants between genetically correlated diseases.

**Fig 3:**
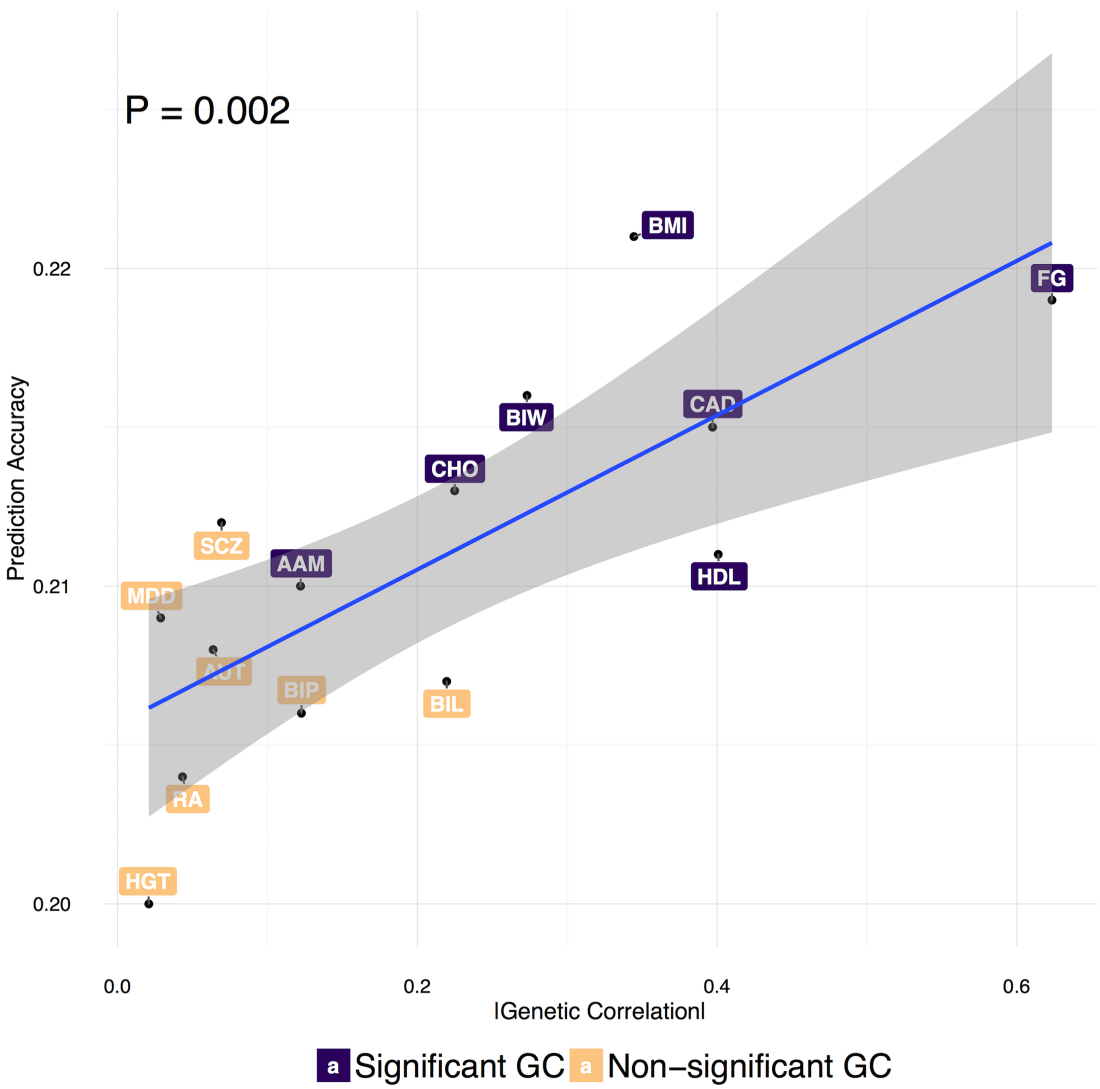
Prediction accuracy of the PleioPred-anno on T2D when jointly modeled with additional traits. Genetic correlations were estimated using LDSC[28] and the significant correlations were labeled in purple. P-value and confidence region indicates the significant correlation between prediction accuracy and genetic correlation. The similar pattern was observed in infinitesimal and non-infinitesimal models without annotations (S1 Fig). AAM: age at menarche, AUT: autism spectrum, BIP: bipolar disorder, BMI: body mass index, BIL: birth length, BIW: birth weight, CHO: childhood obesity, CAD: coronary artery disease, FG: fasting glucose, HDL: HDL Cholesterol, MDD: major depressive disorder, RA: rheumatoid arthritis, and SCZ: schizophrenia.

Correlations between the calculated PRS and disease status (COR) for different approaches and area under the ROC curve (AUC) are summarized in Table 1 and S1 Table. In both infinitesimal and non-infinitesimal models, we observed that two-trait models consistently outperformed single-trait methods and incorporating functional annotations could further improve the prediction accuracy across different diseases. Furthermore, non-infinitesimal models achieved much better performance than infinitesimal models. We also fitted a logistic regression model with the case/control status as outcome and PRS as covariates and reported the corresponding slopes of PRSs, which measures the increase in odds ratio of getting disease with a unit change in PRS (Table 1) and further validated the advantage of integrating pleiotropy and functional annotations. A likelihood ratio test was used to test for the difference in the prediction accuracy between models comparing the likelihood of a logistic regression fitting PRS of one method to that of a logistic regression fitting PRS of two methods jointly (Table 2). From the test, PleioPred with 61 annotations performed significantly better than single-trait models (infinitesimal model: p = 1.4e-33 for CD, p = 1.6e-12 for CEL and p = 1.7e-3 for T2D; non-infinitesimal model: p = 5.2e-29 for CD, p = 2.8e-7 for CEL and p = 0.027 for T2D). Reversing the order of test (that is, comparing the likelihood of two-trait model with that of two-trait and single-trait model jointly or model using annotations with model using and not using annotations jointly) results in non-significant p-values for most tests (S2 Table), which further demonstrates that PRS incorporating functional annotations and pleiotropy mostly encompasses the information of PRS of single trait model. Besides CAD, we also jointly modeled T2D with a spectrum of traits, whose genetic correlations with T2D have been systematically studied [28], including age at menarche (AAM), autism spectrum (AUT), bipolar disorder (BIP), body mass index (BMI), birth length (BIL), birth weight (BIW), childhood obesity (CHO), fasting glucose (FG), HDL Cholesterol (HDL), height (HGT), major depressive disorder (MDD), rheumatoid arthritis (RA) and schizophrenia (SCZ). We estimated the genetic correlations between T2D and these traits using LDSC[21, 28] and showed that the increment in prediction accuracy is significantly correlated with the genetic correlation between T2D and the jointly modeled traits (P = 0.002; Fig 3 and S1 Fig).

**Table 1:** Mean CORs and Regression slopes of infinitesimal and non-infinitesimal methods in independent validation cohort of CE, CEL, and T2D. For two-trait prediction models, we jointly modeled CD with UC, CEL with UC and, T2D with CAD.

	COR^a^			Regression Slope^b^		
	CD	CEL	T2D	CD	CEL	T2D
ldpred-inf	0.196	0.072	0.137	0.454	0.168	1.99
AnnoPred-inf	0.219	0.098	0.145	0.572	0.255	2.15
PleioPred-inf	0.246	0.100	0.168	0.661	0.292	2.198
PleioPred-anno-inf	**0.248**	**0.122**	**0.184**	**0.739**	**0.400**	**2.333**
ldpred	0.247	0.120	0.217	0.873	0.661	2.83
AnnoPred	0.279	0.132	0.219	1.306	0.924	2.86
PleioPred	**0.307**	0.141	**0.225**	1.284	1.332	3.05
PleioPred-anno	0.297	**0.156**	0.22	**1.340**	**1.361**	**3.063**

^a^ correlations between disease status and PRS;

^b^ Regression slopes of logistic regression with case/control status as outcome and PRS as covariates, larger value indicates a larger increase in odds ratio when PRS increases by one unit.

**Table 2:** p-values from the likelihood ratio tests comparing different models.

		CD	CEL	T2D
x_1_	x_2_	p-values from LRT^a^		
**ldpred-inf**	**AnnoPred-inf**	**4.4e-15**	**2.8e-6**	**0.011**
ldpred-inf	PleioPred-inf	3.9e-34	2.3e-7	0.041
AnnoPred-inf	PleioPred-anno-inf	1.5e-18	4.9e-8	0.031
PleioPred-inf	PleioPred-anno-inf	1.8e-9	1.9e-8	0.017
**ldpred-inf**	**PleioPred-anno-inf**	**6.4e-31**	**1.6e-12**	**1.7e-3**
**ldpred**	**AnnoPred**	**1.3e-5**	**1.7e-5**	**0.066**
ldpred	PleioPred	9.3e-40	0.022	0.039
AnnoPred	PleioPred-anno	8.6e-13	5.7e-5	0.021
PleioPred	PleioPred-anno	7.7e-3	0.014	0.45
**ldpred**	**PleioPred-anno**	**5.2e-29**	**2.8e-7**	**0.027**

^a^ Likelihood ratio = -2[logL(x_1_)—logL(x_1_ + x_2_)], where logL(x_1_) and logL(x_1_ + x_2_) is the log likelihood from a logistic regression with case/control status as outcome and x_1_ and x_2_ as covariates.

**Fig 4:**
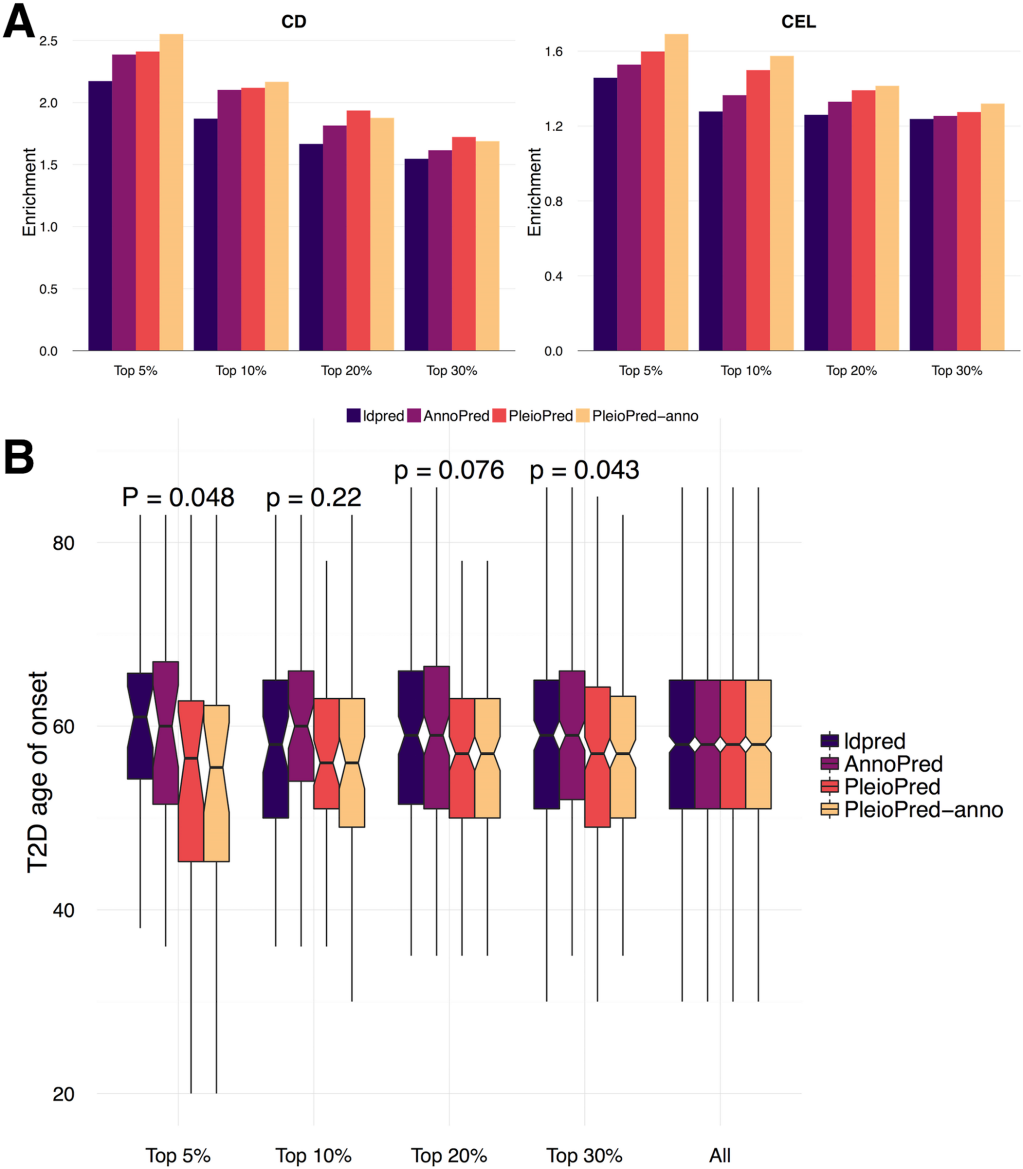
Comparing non-infinitesimal methods in different standards. **(A)** Enrichment of proportion of cases in testing samples with high PRS (top 1%, 5%, 10%, 20% and 30% risk groups stratified by PRS) in CD and CEL. **(B)** Distribution of age of onset of T2D in testing samples with high PRS (top 5%, 10%, 20% and 30% risk groups stratified by PRS) in T2D. P-values were calculated using Wilcoxon rank test comparing the two-trait models with the one-trait models. The last column represents the overall age of onset in testing samples.

Since COR only measures the global discriminating power of prediction method, it might not be the best evaluation metric for risk prediction approaches, with which it is of more use to stratify the population into clinically meaningful groups[1, 17, 29]. In order to test different methods’ ability to stratify individuals with high risk, we compared the proportion of cases among testing samples with high PRS from non-infinitesimal models in CD and CEL. PleioPred-anno showed highest power in stratifying patients within the top risk population (Fig 4A). For T2D, we compared the distribution of the age of onset within risk groups stratified by different non-infinitesimal PRSs (Fig 4B). Onset ages of T2D are significantly lower among the individuals with higher two-trait PRS than those with higher single-trait PRS, which indicates that PRS of two-trait methods could effectively stratify the population with high absolute risk of T2D and demonstrates the potential clinical usage of the PleioPred and the advantage of joint modeling of related diseases over single-trait prediction methods.

In the non-infinitesimal two-trait model, the major contribution to improved performance came from pleiotropy. That is, the variants that are causal in both diseases would be prioritized and those are not causal or have smaller effect sizes in both diseases would be given lower effect size estimation. Therefore, incorporating a genetically correlated disease is equivalent to integrating a functional annotation and its effectiveness and power depend on the genetic correlation between two diseases. When the two diseases are very similar and share a large amount of causal and non-causal variants, adding less effective annotations may dilute the signals and lead to lower prediction accuracy. This aligns with our results in Tables 1 and 2, in which CD-UC and T2D-CAD have a rather high genetic correlation (0.427, 0.432 respectively) and PleioPred yields better performance. On the contrary, CEL-UC have a relatively lower genetic correlation (0.283) and PleioPred-anno yields the best prediction accuracy. We performed further analysis with T2D and 13 other correlated diseases (those used in Fig 3). We plot the prediction accuracy of PleioPred and PleioPred-anno against absolute genetic correlation and it can be seen that when the functional annotations are fixed, as the absolute genetic correlation increases, PleioPred tends to yield slightly better results (S2 Fig).

## Discussion

Our work demonstrates that pleiotropy and functional annotations can effectively improve the performance of genetic risk prediction. PleioPred jointly analyzes genetically correlated diseases and diverse types of annotation data with GWAS summary statistics to upweight causal SNPs shared between diseases and with a higher likelihood of functionality, which lead to consistently better prediction accuracy for multiple complex diseases. Besides prediction accuracy, PleioPred can better stratify population into different risk groups and has greater potential in clinical usage. Our method is not without limitation. First, despite consistent improvement compared with existing PRS-based methods, AUCs for most diseases remain moderate. In order to effectively stratify risk groups for clinical usage, our model remains to be further calibrated using large cohorts with measured environmental and clinical risk factors [1]. Second, accurate estimation of GWAS signal enrichment and SNP effect sizes requires a large sample size for the training dataset. This could be potentially improved by better estimators for annotation-stratified heritability in the future [30]. Third, it is non-trivial to foresee whether PleioPred or PleioPred-anno would work better for a given pair of diseases. According to our observation in real data analysis, PleioPred would eventually outperform PleioPred-anno with an increasing genetic correlation. The threshold at which the change happens could be learned with a validation dataset in practice. The proposed framework can be easily customized and extended to incorporate more than two diseases, which could potentially further increase the prediction accuracy. However, it is worth noting that computation burden and the difficulty in model fitting also increases with the number of diseases. Furthermore, many GWAS have shared control samples, which may result in duplicated information and noise in the training samples. A few Bayesian models combining GWAS summary statistics with functional annotations have been proposed for the purpose of fine-mapping functional variants [31–33]. Whether these models could be adapted to benefit risk prediction accuracy remains to be investigated in the future. Importantly, the rich collection of publicly available integrative annotation data, in conjunction with the increasing accessibility of GWAS summary statistics, makes PleioPred a customizable and powerful tool. As GWAS sample size continues to grow, PleioPred has the potential to achieve even better prediction accuracy and become widely adopted as a summary of genetic contribution in clinical applications of risk prediction. Although more and more GWAS summary results are becoming available [34], in order to evaluate the prediction accuracy, a cohort independent with both training GWAS samples is required, which is very challenging to find. We will apply the proposed methods to a wide range of diseases when independent validation data become available in the future.

## Methods

### Conditional distribution of marginal effect size estimators

Assume the phenotypes of two diseases $Y_{N_{1}\times 1}^{\left(1\right)},\;Y_{N_{2}\times 1}^{\left(2\right)}$ and the genotypes $X_{N_{1}\times M},\;Z_{N_{2}\times M}$ are standardized with mean zero and variance one. Here N_1_ and N_2_ denote the sample sizes for the two diseases and M is the number of markers. We further assume a linear model with genotype matrices, effect sizes (*β* and *γ*) and random errors (*ε* and *δ*) mutually independent.

$$Y_{N_{1}\times 1}^{\left(1\right)}=X_{N_{1}\times M}\beta_{M\times 1}+\varepsilon_{N_{1}\times 1}$$

$$Y_{N_{2}\times 1}^{\left(2\right)}=Z_{N_{2}\times M}\gamma_{M\times 1}+\delta_{N_{2}\times 1}$$

Assume that the effect sizes of different SNPs are independent. As for random errors, we assume that

$$\left(\begin{array}{c} \varepsilon \\ \delta \end{array}\right)\sim N\left(\left(\begin{array}{c} 0\\ 0 \end{array}\right),\left[\begin{array}{cc} \left(1-h_{1}^{2}\right)I_{N_{1}} & S\\ S^{T} & \left(1-h_{2}^{2}\right)I_{N_{2}} \end{array}\right]\right)$$

$$S_{ij}=\{\begin{array}{ll} \rho_{e} & \text{if individual i in study }1\text{ and individual j in study }2\text{ are the same}\\ 0 & \text{if individual i in study }1\text{ and individual j in study }2\text{ are different} \end{array}$$

where $h_{1}^{2}$ and $h_{2}^{2}$ denote the heritability of two diseases and *ρ*_*e*_ measures the covariance within the overlapping individuals between two studies. Denote the LD matrix and marginal effect size estimator from GWAS as: ${\hat{D}}_{1}=\frac{1}{N_{1}}X^{T}X,\;\;{\hat{D}}_{2}=\frac{1}{N_{2}}Z^{T}Z,\;\;\tilde{\beta }=\frac{1}{N_{1}}X^{T}Y^{\left(1\right)}$ and $\tilde{\gamma }=\frac{1}{N_{2}}Z^{T}Y^{\left(2\right)}$. In practice, ${\hat{D}}_{1}$ and ${\hat{D}}_{2}$ can be estimated from a reference panel and we therefore denote the LD matrix as $\hat{D}$ for convenience. Then following the derivation in [13], we can derive the conditional distribution of GWAS summary statistics as

$$\begin{array}{c} \tilde{\beta }\\ \tilde{\gamma } \end{array}|\begin{array}{c} \beta \\ \gamma \end{array},\hat{D}\sim N\left(\left(\begin{array}{c} \hat{D}\beta \\ \hat{D}\gamma \end{array}\right),\;\left[\begin{array}{cc} \frac{1-h_{1}^{2}}{N_{1}}\hat{D} & \frac{N_{S}\rho_{e}}{N_{1}N_{2}}\hat{D}\\ \frac{N_{S}\rho_{e}}{N_{1}N_{2}}\hat{D} & \frac{1-h_{2}^{2}}{N_{2}}\hat{D} \end{array}\right]\right)$$

where *N*_*s*_ is the number of overlapping samples between the two studies. When *N*_*s*_ is relatively small, we can discard terms with $\frac{N_{S}\rho_{e}}{N_{1}N_{2}}$ to reduce the computation burden. In practice, we usually ignore the overlap between samples mainly due to four reasons: 1) it is usually challenging to estimate the parameter *ρ*_*e*_ and obtain the exact number of overlapping samples. 2) The off-diagonal term $\frac{N_{S}\rho_{e}}{N_{1}N_{2}}$ is much smaller comparing to the diagonal terms ($\frac{N_{S}}{N_{1}N_{2}}\sim \frac{1}{N_{1}^{2}}$). Even in the case of complete overlap where $\frac{N_{S}\rho_{e}}{N_{1}N_{2}}=\frac{\rho_{e}}{N_{1}}$, *ρ*_*e*_ is still at the magnitude of $\left(1-h_{1}^{2}\right)\left(1-h_{2}^{2}\right)$. 3) sensitivity analysis through simulations indicated that the method is very robust to overlapping samples (S6 Table). 4) In practice, *ρ*_*e*_ can be estimated via LDSC if *N*_*S*_ is known. However, including the covariance matrix of $\tilde{\beta }$ and $\tilde{\gamma }$ can significantly increase the computational cost and thus increase the variability of estimation.

### Infinitesimal model

Assume that the effect sizes follow a multivariate normal distribution:

$$\left(\begin{array}{c} \beta \\ \gamma \end{array}\right)\sim N\left(\left(\begin{array}{c} 0\\ 0 \end{array}\right),\left[\begin{array}{cc} diag\left(\sigma_{1i}^{2}\right) & \rho_{g}*diag\left(\sigma_{1i}\sigma_{2i}\right)\\ \rho_{g}*diag\left(\sigma_{1i}\sigma_{2i}\right) & diag\left(\sigma_{2i}^{2}\right) \end{array}\right]\right)$$

where $\sigma_{1i}^{2}$ and $\sigma_{2i}^{2}$ denote the variance of effect sizes of SNP *i* and *ρ*_*g*_: = *cor*(*β*_*i*_, *γ*_*i*_), representing the genetic correlation between two diseases. Suppose that the whole genome is partitioned into *K* functional regions *A*_1_,…, *A*_*K*_. Specific annotations used in PleioPred were described previously (Results). We assume the effect size of a SNP depends on the functional regions it falls in and the effect sizes are additive in the overlapping regions:

$$var\left(\beta_{i}\right)=\sigma_{1i}^{2}=\;\sum_{c:i\in A_{c}}\tau_{1c}$$

$$var\left(\gamma_{i}\right)=\sigma_{2i}^{2}=\;\sum_{c:i\in A_{c}}\tau_{2c}$$

where *τ*_*jc*_ denotes the variance of the effect size of SNPs on disease *j* falling in *A*_*c*_ alone.

For parameter estimation, we applied a two-stage approach: first,$\sigma_{1i}^{2}$ and $\sigma_{2i}^{2}$ are estimated using annotation stratified LD score regression (LDSC)[21], which is essentially a method of moments estimator since LDSC utilizes the relationship between the second moment of marginal estimators and variance components of each functional region.

$$EN_{1}{\tilde{\beta }}_{i}^{2}\approx N_{1}\underset{c}{\sum }\tau_{1c}l\left(i,c\right)+1$$

$$EN_{2}{\tilde{\gamma }}_{i}^{2}\approx N_{2}\underset{c}{\sum }\tau_{2c}l\left(i,c\right)+1$$

$$l\left(i,c\right)=\sum_{k\in A_{c}}{\left(EX_{i}X_{k}\right)}^{2}$$

Specifically for each disease, we use ${\hat{\sigma }}_{ji}^{2}=C_{j}\left(\sum_{c:\;i\in A_{c}}{\hat{\tau }}_{jc}\right)$ to specify the per-SNP heritability for disease *j* where *C*_*j*_ is a constant calculated from the following equation

$$\sum_{i}{\hat{\sigma }}_{ji}^{2}={\hat{H}}_{j}^{2}$$

We do not directly use $\sum_{c:\;i\in A_{c}}{\hat{\tau }}_{jc}$ as the per-SNP heritability because it is estimated in the context where all SNPs in the 1000 Genomes database are included in the model [21]. Such per-SNP heritability estimates cannot be extrapolated to the risk prediction context where many fewer SNPs are analyzed [35]. Therefore, we rescale the heritability estimates to better quantify each SNP’s contribution towards chip heritability. Following [36], we use a summary statistics-based heritability estimator that approximates the Haseman-Elston estimator:

$${\hat{H}}_{j}^{2}=\frac{\left({\bar{\chi }}_{j}^{2}-1\right)}{N_{j}\bar{l}}$$

where ${\bar{\chi }}_{j}^{2}$ and $\bar{l}$ denote the mean squared marginal estimators ($N_{1}{\tilde{\beta }}_{i}^{2}$ and $N_{2}{\tilde{\gamma }}_{i}^{2}$ for diseases 1 and 2) and the mean non-stratified LD score, respectively.

In the GWAS setting, $\hat{D}$ are usually non-invertible and have very high dimensions. We thus study the posterior distribution of a small chunk of marginal effect size estimators instead. Let ${\tilde{\beta }}_{b}$ and ${\tilde{\gamma }}_{b}$ be the estimated marginal effect sizes of SNPs in a region *b* (e.g. an LD block) and the corresponding genotype matrices are *X*_*b*_ and *Z*_*b*_ and sample correlation matrices is ${\hat{D}}_{b}$, respectively. Then the conditional distribution of the marginal effect size estimators is (assuming no overlapping individuals or omitting the off-diagonal terms)

$$\begin{array}{c} {\tilde{\beta }}_{b}\\ {\tilde{\gamma }}_{b} \end{array}|\begin{array}{c} \beta_{b}\\ \gamma_{b} \end{array},{\hat{D}}_{b}\sim N\left(\left(\begin{array}{c} {\hat{D}}_{b}\beta_{b}\\ {\hat{D}}_{b}\gamma_{b} \end{array}\right),\;\left[\begin{array}{cc} \frac{1-h_{1b}^{2}}{N_{1}}{\hat{D}}_{b} & 0\\ 0 & \frac{1-h_{2b}^{2}}{N_{2}}{\hat{D}}_{b} \end{array}\right]\right)$$

$h_{1b}^{2}=\sum_{i\;\in b}\sigma_{1i}^{2}$ and $h_{2b}^{2}=\sum_{i\;\in b}\sigma_{2i}^{2}$ denote the heritability of SNPs in region *b* for the two diseases, which are usually close to zero since the region *b* is relatively small and can be safely rounded to zero in calculation. We choose the size of *b* using the standard described in [9].

Finally, we treat *ρ*_*e*_ as a tuning parameter and the posterior expectation of the effect sizes can be calculated as:

$$E\left(\begin{array}{c} \beta_{b}\\ \gamma_{b} \end{array}|\begin{array}{c} {\tilde{\beta }}_{b}\\ {\tilde{\gamma }}_{b} \end{array},{\hat{D}}_{b}\right)\approx {\left(\left[\begin{array}{cc} N_{1}{\hat{D}}_{b} & 0\\ 0 & N_{2}{\hat{D}}_{b} \end{array}\right]+{\left[\begin{array}{cc} diag\left(\sigma_{1i}^{2}\right) & \rho_{g}*diag\left(\sigma_{1i}\sigma_{2i}\right)\\ \rho_{g}*diag\left(\sigma_{1i}\sigma_{2i}\right) & diag\left(\sigma_{2i}^{2}\right) \end{array}\right]}^{-1}\right)}^{-1}\left(\begin{array}{c} N_{1}{\tilde{\beta }}_{b}\\ N_{2}{\tilde{\gamma }}_{b} \end{array}\right)$$

### Non-infinitesimal model

In practice[9, 13], we note that a sparse model yields a higher accuracy for most diseases. Moreover, the infinitesimal model assumption is relatively strong in some cases. For example, two related diseases may only share some causal variants and have no correlation among the effect sizes or the correlation structures may vary across the genome. We therefore propose a hierarchical Bayesian model with a more general assumption and we refer to this framework as the non-infinitesimal model. Under this model, we assume that the effect sizes follow a mixed distribution.

$$\left(\begin{array}{c} \beta_{i}\\ \gamma_{i} \end{array}\right)|\vec{p}≜\left(p_{11},p_{10},p_{01},p_{00}\right)\sim p_{11}\left(\begin{array}{c} N\left(0,\frac{\sigma_{1i}^{2}}{p_{11}+p_{10}}\right)\\ N\left(0,\frac{\sigma_{2i}^{2}}{p_{11}+p_{01}}\right) \end{array}\right)+p_{10}\left(\begin{array}{c} N\left(0,\frac{\sigma_{1i}^{2}}{p_{11}+p_{10}}\right)\\ \delta_{0} \end{array}\right)+p_{01}\left(\begin{array}{c} \delta_{0}\\ N\left(0,\frac{\sigma_{2i}^{2}}{p_{11}+p_{01}}\right) \end{array}\right)+p_{00}\left(\begin{array}{c} \delta_{0}\\ \delta_{0} \end{array}\right)$$

$$\vec{p}\sim Dirichlet\left(\alpha \right)$$

That is, the effect sizes of SNP *i* to two diseases follow a mixed distribution with normal (when SNP *i* is causal in both diseases), joint normal and point mass (when SNP *i* is causal in only one diseases) and joint point mass (when SNP *i* is not causal in either disease). Although we do not have closed form solution for the posterior expectation of the effect sizes, we can use Gibbs sampler to sample from the posterior distribution of the effect sizes to estimate the posterior expectation. The joint posterior distribution of *β*_*i*_ and *γ*_*i*_ given $\tilde{\beta }$, $\tilde{\gamma }$, *β*_−*i*_, *γ*_−*i*_ and $\vec{p}$ is

$$\begin{array}{l} f\left(\beta_{i},\gamma_{i}|\tilde{\beta },\tilde{\gamma },\beta_{-i},\;\gamma_{-i},\;\vec{p},\hat{D},\sigma_{1i}^{2},\sigma_{2i}^{2}\right)\\ \propto p_{11}\sqrt{\frac{1}{N_{1}\frac{\sigma_{1i}^{2}}{p_{11}+p_{10}}+1}}\sqrt{\frac{1}{N_{2}\frac{\sigma_{2i}^{2}}{p_{11}+p_{01}}+1}}\left(\begin{array}{c} N(C_{1}\Delta_{1},\frac{C_{1}}{N_{1}})\\ N(C_{2}\Delta_{2},\frac{C_{2}}{N_{2}}) \end{array}\right)\\ +p_{10}\sqrt{\frac{1}{N_{1}\frac{\sigma_{1i}^{2}}{p_{11}+p_{10}}+1}}\text{exp}\left(-\frac{N_{1}}{2}C_{1}\Delta_{1}^{2}\right)\left(\begin{array}{c} N\left(C_{1}\Delta_{1},\;\frac{C_{1}}{N_{1}}\right)\\ \delta_{0} \end{array}\right)\\ +p_{01}\sqrt{\frac{1}{N_{2}\frac{\sigma_{2i}^{2}}{p_{11}+p_{01}}+1}}\text{exp}\left(-\frac{N_{2}}{2}C_{2}\Delta_{2}^{2}\right)\left(\begin{array}{c} \delta_{0}\\ N\left(C_{2}\Delta_{2},\frac{C_{2}}{N_{2}}\right) \end{array}\right)\\ +p_{00}\text{exp}\left(-\frac{N_{1}}{2}C_{1}\Delta_{1}^{2}\right)\text{exp}\left(-\frac{N_{2}}{2}C_{2}\Delta_{2}^{2}\right)\left(\begin{array}{c} \delta_{0}\\ \delta_{0} \end{array}\right) \end{array}$$

$$\beta_{i},\gamma_{i}|\tilde{\beta },\tilde{\gamma },\beta_{-i},\;\gamma_{-i},\;\vec{p},\hat{D},\sigma_{1i}^{2},\sigma_{2i}^{2}\sim {\tilde{p}}_{11}\left(\begin{array}{c} N(C_{1}\Delta_{1},\frac{C_{1}}{N_{1}})\\ N(C_{2}\Delta_{2},\frac{C_{2}}{N_{2}}) \end{array}\right)+{\tilde{p}}_{10}\left(\begin{array}{c} N\left(C_{1}\Delta_{1},\;\frac{C_{1}}{N_{1}}\right)\\ \delta_{0} \end{array}\right)+{\tilde{p}}_{01}\left(\begin{array}{c} \delta_{0}\\ N\left(C_{2}\Delta_{2},\frac{C_{2}}{N_{2}}\right) \end{array}\right)+{\tilde{p}}_{00}\left(\begin{array}{c} \delta_{0}\\ \delta_{0} \end{array}\right)$$

$$C_{1}≜\frac{N_{1}}{N_{1}+\frac{p_{11}+p_{10}}{\sigma_{1i}^{2}}},C_{2}≜\frac{N_{2}}{N_{2}+\frac{p_{11}+p_{01}}{\sigma_{2i}^{2}}},\;\;\Delta_{1}≜{\hat{\beta }}_{i}-\sum_{j\neq i}{\hat{D}}_{ij}\beta_{j},\;\;\Delta_{2}≜{\hat{\gamma }}_{i}-\sum_{j\neq i}{\hat{D}}_{ij}\gamma_{j}$$

$${\tilde{p}}_{11}=p_{11}\sqrt{\frac{1}{N_{1}\frac{\sigma_{1i}^{2}}{p_{11}+p_{10}}+1}}\sqrt{\frac{1}{N_{2}\frac{\sigma_{2i}^{2}}{p_{11}+p_{01}}+1}}/p_{sum}$$

$${\tilde{p}}_{10}=p_{10}\sqrt{\frac{1}{N_{1}\frac{\sigma_{1i}^{2}}{p_{11}+p_{10}}+1}}\text{exp}\left(-\frac{N_{1}}{2}C_{1}\Delta_{1}^{2}\right)/p_{sum}$$

$${\tilde{p}}_{01}=p_{01}\sqrt{\frac{1}{N_{2}\frac{\sigma_{2i}^{2}}{p_{11}+p_{01}}+1}}\text{exp}\left(-\frac{N_{2}}{2}C_{2}\Delta_{2}^{2}\right)/p_{sum}$$

$${\tilde{p}}_{00}=p_{00}\text{exp}\left(-\frac{N_{1}}{2}C_{1}\Delta_{1}^{2}\right)\text{exp}\left(-\frac{N_{2}}{2}C_{2}\Delta_{2}^{2}\right)/p_{sum}$$

$$\begin{array}{l} p_{sum}=p_{11}\sqrt{\frac{1}{N_{1}\frac{\sigma_{1i}^{2}}{p_{11}+p_{10}}+1}}\sqrt{\frac{1}{N_{2}\frac{\sigma_{2i}^{2}}{p_{11}+p_{01}}+1}}\\ +p_{10}\sqrt{\frac{1}{N_{1}\frac{\sigma_{1i}^{2}}{p_{11}+p_{10}}+1}}\text{exp}\left(-\frac{N_{1}}{2}C_{1}\Delta_{1}^{2}\right)\\ +p_{01}\sqrt{\frac{1}{N_{2}\frac{\sigma_{2i}^{2}}{p_{11}+p_{01}}+1}}\text{exp}\left(-\frac{N_{2}}{2}C_{2}\Delta_{2}^{2}\right)\\ +p_{00}\text{exp}\left(-\frac{N_{1}}{2}C_{1}\Delta_{1}^{2}\right)\text{exp}\left(-\frac{N_{2}}{2}C_{2}\Delta_{2}^{2}\right) \end{array}$$

The posterior distribution of $\vec{p}$ is rather complicated and we therefore applied a Metropolis Hastings method to sample $\vec{p}$ and use the following proposing distribution.

$$\vec{p}\sim Dirichlet\left(\left(\alpha_{1}+d_{11},\;\alpha_{2}+d_{10},\;\alpha_{3}+d_{01},\;\alpha_{4}+d_{00}\right)\right)$$

in which *d*_11_ represents the number of SNPs that are causal in both diseases, *d*_10_ and *d*_01_ represent the number of SNPs that are causal in only one disease and *d*_00_ denotes the number of non-causal SNPs from previous sampling step. To ensure convergence, we shrink the posterior probability of being causal if the estimation of heritability at current step of either disease is larger than the heritability estimated from the GWAS summary statistics. That is, $\left({\tilde{p}}_{11},{\tilde{p}}_{10},{\tilde{p}}_{01}\right)$ are shrunken by a factor $c=\text{min}\left(1,\frac{{\hat{h}}_{1}^{2}}{\sum_{j}{\hat{\beta }}_{\left(i\right),j}^{2}},\frac{{\hat{h}}_{2}^{2}}{\sum_{j}{\hat{\gamma }}_{\left(i\right),j}^{2}}\right)$, where ${\hat{\beta }}_{\left(i\right),j}$ and ${\hat{\gamma }}_{\left(i\right),j}$ are the sampled effect size of SNP *j* in the *i*th iteration. And simulations showed the algorithm yields fast convergence and high accuracy in estimation (S5 Table). An important advantage about the non-infinitesimal approach is that it has no tuning parameters and thus more computationally efficient. Furthermore, by imposing a Bayesian shrinkage, we can better select functionally relevant variants and tune down the unrelated information.

The running time mainly depends on the number of SNPs and iterations in MCMC steps used in prediction and for a typical GWAS dataset with 400,000 SNPs, it usually takes approximately two hours to finish 250 iterations in MCMC (which already leads to good convergence). And we recommend using at least one thousand unrelated individuals with the same ancestry for which summary statistics datasets are obtained from following the same guideline of [9].

### Ethics statement

The study was approved by YALE UNIVERSITY HUMAN INVESTIGATION COMMITTEE with approval number 100 FR1 and 100 FR27.

### Software availability

PleioPred software: https://github.com/yiminghu/PleioPred

AnnoPred software: https://github.com/yiminghu/AnnoPred

GenoCanyon: http://genocanyon.med.yale.edu/

GenoSkyline: http://genocanyon.med.yale.edu/GenoSkyline

### Supplemental data

Supplemental data include two figures and six tables and detailed description of GWAS summary statistics and validation cohorts.

## Supporting information

S1 Fig Prediction accuracy of the PleioPred-inf, PleioPred-anno-inf and PleioPred on T2D when jointly modeled with a wide spectrum of diseases. Genetic correlations were estimated using LDSC[28] and significant correlations were labeled in purple. P value and confidence region indicates the significant correlation between increment in prediction accuracy and genetic correlation. AAM: age at menarche, AUT: autism spectrum, BIP: bipolar disorder, BMI: body mass index, BIL: birth length, BIW: birth weight, CHO: childhood obesity, CAD: coronary artery disease, FG: fasting glucose, HDL: HDL Cholesterol, MDD: major depressive disorder, RA: rheumatoid arthritis and SCZ: schizophrenia.
(TIFF)

S2 Fig Prediction accuracy of the PleioPred and PleioPred-anno on T2D when jointly modeled with a wide spectrum of diseases. Genetic correlations were estimated using LDSC[28]. (AAM: age at menarche, gc (genetic correlation) = 0.1221; AUT: autism spectrum, gc = 0.0638; BIP: bipolar disorder, gc = 0.1227; BMI: body mass index, gc = 0.3445; BIL: birth length, gc = 0.2196; BIW: birth weight, gc = 0.2732; CHO: childhood obesity, gc = 0.2249; CAD: coronary artery disease, gc = 0.432; FG: fasting glucose, gc = 0.6234; HDL: HDL Cholesterol, gc = 0.4008; MDD: major depressive disorder, gc = 0.0288; RA: rheumatoid arthritis, gc = 0.0434; and SCZ: schizophrenia, gc = 0.0694).
(TIFF)

S1 Table Mean AUCs of infinitesimal and non-infinitesimal methods in independent validation cohort of CE, CEL, and T2D. For two-trait prediction models, we jointly modeled CD with UC, CEL with UC, and T2D with CAD.
(XLSX)

S2 Table p-values from the likelihood ratio tests comparing different models. (XLSX)

S3 Table URLs of GWAS summary statistics. (XLSX)

S4 Table URLs of validation data. (XLSX)

S5 Table Accuracy of parameter estimation in simulations using the proposed MCMC method. Data were generated from real genotype data of chromosome 1 with 29,596 individuals for both traits. We random selected 300 out of 41,334 SNPs as causal variants with 1/3 shared between two traits. We simulated in total 6 scenarios corresponding to different heritability of two traits. In each setting we use the maximum of mean squared error (MAX_MSE) of effect sizes of 41,334 SNPs to evaluate the estimation accuracy.
(XLSX)

S6 Table Influence of overlapped individuals in training samples. Data were generated from real genotype data of chromosome 1 with 29,596 individuals for both traits. We random selected 300 out of 41,334 SNPs as causal variants with 1/3 shared causal variants. N_s: the number of overlapping individuals between diseases; rho_e: the covariance between random errors of two traits on the same individuals (see Methods for details); MAX_MSE1 and MAX_MSE2: the maximum of mean squared error of effect sizes of 41,334 SNPs in two traits respectively (100 replications, used for evaluating estimation accuracy).
(XLSX)

S7 Table Mean AUCs of MTAG compared with other methods in real data analysis. (XLSX)

S1 Text Details on GWAS summary statistics and validation data. (DOCX)
