# Supplementary figures and images for: Joint modeling of genetically correlated diseases and functional annotations increases accuracy of polygenic risk prediction
Source: PLoS Genet. 2017 Jun 9;13(6):e1006836. doi: 10.1371/journal.pgen.1006836 (PMC5482506; doi:10.1371/journal.pgen.1006836)

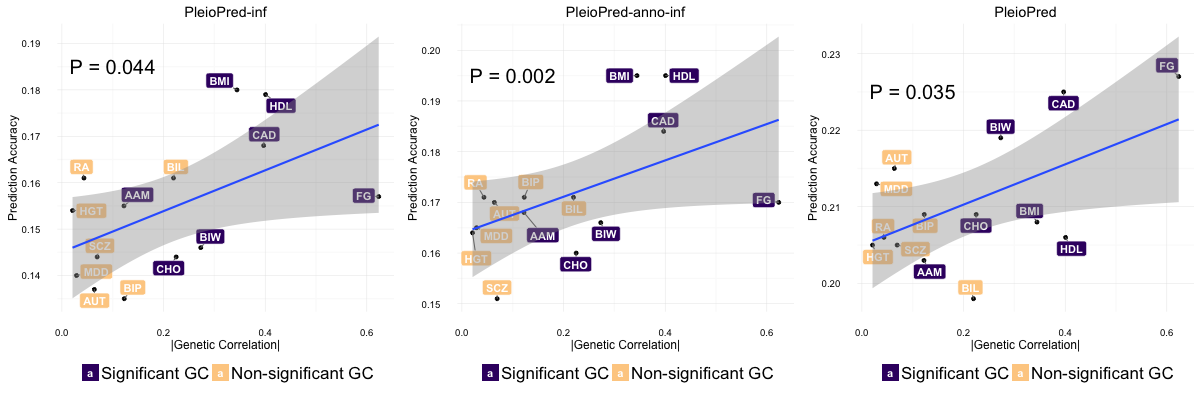

Supplement: S1 Fig — Genetic correlations were estimated using LDSC[28] and significant correlations were labeled in purple. P value and confidence region indicates the significant correlation between increment in prediction accuracy and genetic correlation. AAM: age at menarche, AUT: autism spectrum, BIP: bipolar disorder, BMI: body mass index, BIL: birth length, BIW: birth weight, CHO: childhood obesity, CAD: coronary artery disease, FG: fasting glucose, HDL: HDL Cholesterol, MDD: major depressive disorder, RA: rheumatoid arthritis and SCZ: schizophrenia. (TIFF) [file pgen.1006836.s001.tiff]

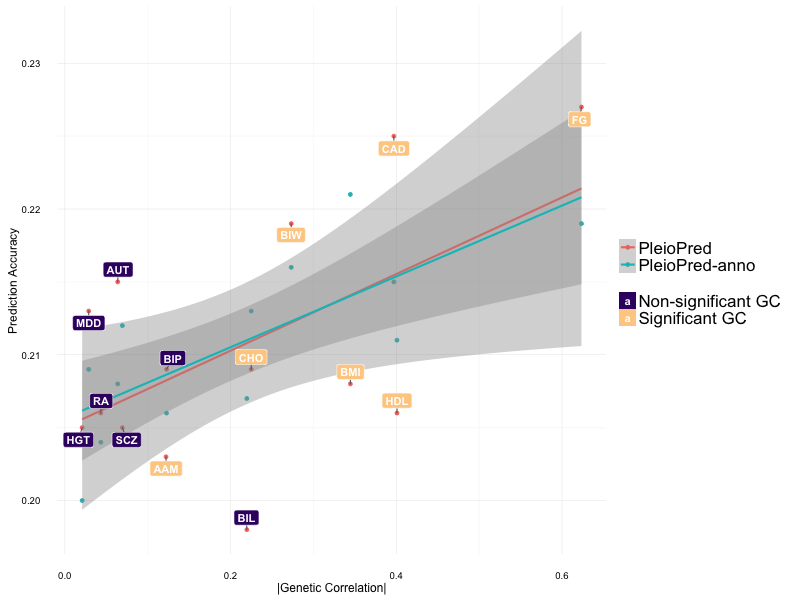

Supplement: S2 Fig — Genetic correlations were estimated using LDSC[28]. (AAM: age at menarche, gc (genetic correlation) = 0.1221; AUT: autism spectrum, gc = 0.0638; BIP: bipolar disorder, gc = 0.1227; BMI: body mass index, gc = 0.3445; BIL: birth length, gc = 0.2196; BIW: birth weight, gc = 0.2732; CHO: childhood obesity, gc = 0.2249; CAD: coronary artery disease, gc = 0.432; FG: fasting glucose, gc = 0.6234; HDL: HDL Cholesterol, gc = 0.4008; MDD: major depressive disorder, gc = 0.0288; RA: rheumatoid arthritis, gc = 0.0434; and SCZ: schizophrenia, gc = 0.0694). (TIFF) [file pgen.1006836.s002.tiff]
