# Supplementary material for: Joint modeling of genetically correlated diseases and functional annotations increases accuracy of polygenic risk prediction
Source: PLoS Genet. 2017 Jun 9;13(6):e1006836. doi: 10.1371/journal.pgen.1006836 (PMC5482506; doi:10.1371/journal.pgen.1006836)
Supplement: S1 Text — (DOCX) [file pgen.1006836.s010.docx]

**Supplemental Texts**

**Details on GWAS summary statistics and validation data**

For Crohn’s disease, we used the International Inflammatory Bowel Disease Genetics Consortium (IIBDGC) summary statistics (6,333 Crohn’s disease patients and 15,056 controls)[1] and (6,687 Ulcerative Colitis (UC) disease patients and 19,718 controls)[2]. WTCCC was removed from the meta-analysis[3]. We used WTCCC cases and GERA (dbGaP access number phs000674.v1.p1) controls as validation set since WTCCC controls were used in the Training data of UC. Genotypes were imputed using IMPUTE2[4, 5] with the 1000 Genomes Project data as reference and only SNPs with info scores larger than 0.5 were kept for further analysis. We filtered individuals with genetic relatedness larger than 0.05 and SNPs with a missing rate larger than 1% and a minor allele frequency less than 1%. In addition, we filtered SNPs with ambiguous nucleotides and kept SNPs matched the summary statistics by both rs number and alleles. Since testing cases and controls are from different studies and array platform, we thus perform a more stringent quality control. We filtered SNPs with p-values larger than 0.5 in the training data. After QC, the testing cohort consisted of 1,689 cases and 5,488 controls with 54,889 SNPs overlapping the summary statistics.

For celiac disease, we used a GWAS consisting of 4,533 cases and 10,750 controls [6] and IIBDGC (6,687 Ulcerative Colitis disease patients and 19,718 controls). The National Institute of Diabetes and Digestive and Kidney Diseases (NIDDK) celiac disease cases and GERA controls (NIDDK controls overlapped with UC training data) were used as validation data[7]. We performed the same QC as CD due to merging different studies and data from different platforms. After QC, it consisted of 1,716 cases and 5,488 controls with 62,391 SNPs in common.

For type-II diabetes, the training data includes GWAS summary statistics from the Diabetes Genetics Replication and Meta-analysis (DIAGRAM) consortium with 12,171 cases and 56,862 controls[8], Reproductive Genetics Consortium (ReproGen, age at menarche, N=132,989)[9], Psychiatric Genomics Consortium (PGC, autism spectrum, N=10,610; bipolar disorder, N=16,731; major depressive disorder, N=18,759; schizophrenia, N=32,143)[10-12], Early Growth Genetics Consortium (EGG, birth length, N=28,459; birth weight, N=143,677; childhood obesity, N=13,848)[13-15], the Genetic Investigation of Anthropometric Traits consortium (GIANT, height, N=253,288; BMI, N=234,069)[16], the Coronary ARtery DIsease Genome wide Replication and Meta-analysis (CARDIoGRAM) with 22,233 cases and 64,762 controls[17], Meta-Analyses of Glucose and Insulin-related traits Consortium (MAGIC, fasting glucose, N=46186)[18] and Global Lipids Genetic Consortium (GLGC, HDL-Cholesterol, N=99,900)[19] and a meta-analysis of Rheumatoid Arthritis with 5,539 cases and 20,169 controls[6]. For testing data, we used Northwestern NUgene Project and after QC it consisted of 662 cases and 517 controls with 475,629 SNPs in common[20].

**References**

1. Franke A, McGovern DP, Barrett JC, Wang K, Radford-Smith GL, Ahmad T, et al. Genome-wide meta-analysis increases to 71 the number of confirmed Crohn's disease susceptibility loci. Nat Genet. 2010;42(12):1118-25. doi: 10.1038/ng.717. PubMed PMID: 21102463; PubMed Central PMCID: PMCPMC3299551.

2. Anderson CA, Boucher G, Lees CW, Franke A, D'Amato M, Taylor KD, et al. Meta-analysis identifies 29 additional ulcerative colitis risk loci, increasing the number of confirmed associations to 47. Nat Genet. 2011;43(3):246-52. doi: 10.1038/ng.764. PubMed PMID: 21297633; PubMed Central PMCID: PMCPMC3084597.

3. Burton PR, Clayton DG, Cardon LR, Craddock N, Deloukas P, Duncanson A, et al. Genome-wide association study of 14,000 cases of seven common diseases and 3,000 shared controls. Nature. 2007;447(7145):661-78.

4. Howie BN, Donnelly P, Marchini J. A flexible and accurate genotype imputation method for the next generation of genome-wide association studies. PLoS Genet. 2009;5(6):e1000529. doi: 10.1371/journal.pgen.1000529. PubMed PMID: 19543373; PubMed Central PMCID: PMCPMC2689936.

5. Howie B, Marchini J, Stephens M. Genotype imputation with thousands of genomes. G3 (Bethesda). 2011;1(6):457-70. doi: 10.1534/g3.111.001198. PubMed PMID: 22384356; PubMed Central PMCID: PMCPMC3276165.

6. Stahl EA, Raychaudhuri S, Remmers EF, Xie G, Eyre S, Thomson BP, et al. Genome-wide association study meta-analysis identifies seven new rheumatoid arthritis risk loci. Nature genetics. 2010;42(6):508-14.

7. Garner C, Ahn R, Ding YC, Steele L, Stoven S, Green PH, et al. Genome-wide association study of celiac disease in North America confirms FRMD4B as new celiac locus. PLoS One. 2014;9(7):e101428. Epub 2014/07/08. doi: 10.1371/journal.pone.0101428. PubMed PMID: 24999842; PubMed Central PMCID: PMCPmc4084811.

8. Morris AP, Voight BF, Teslovich TM, Ferreira T, Segre AV, Steinthorsdottir V, et al. Large-scale association analysis provides insights into the genetic architecture and pathophysiology of type 2 diabetes. Nature genetics. 2012;44(9):981.

9. Perry JR, Day F, Elks CE, Sulem P, Thompson DJ, Ferreira T, et al. Parent-of-origin-specific allelic associations among 106 genomic loci for age at menarche. Nature. 2014;514(7520):92-7. doi: 10.1038/nature13545. PubMed PMID: 25231870; PubMed Central PMCID: PMCPMC4185210.

10. Major Depressive Disorder Working Group of the Psychiatric GC, Ripke S, Wray NR, Lewis CM, Hamilton SP, Weissman MM, et al. A mega-analysis of genome-wide association studies for major depressive disorder. Mol Psychiatry. 2013;18(4):497-511. doi: 10.1038/mp.2012.21. PubMed PMID: 22472876; PubMed Central PMCID: PMCPMC3837431.

11. Psychiatric GCBDWG. Large-scale genome-wide association analysis of bipolar disorder identifies a new susceptibility locus near ODZ4. Nat Genet. 2011;43(10):977-83. doi: 10.1038/ng.943. PubMed PMID: 21926972; PubMed Central PMCID: PMCPMC3637176.

12. Ripke S, O'Dushlaine C, Chambert K, Moran JL, Kahler AK, Akterin S, et al. Genome-wide association analysis identifies 13 new risk loci for schizophrenia. Nat Genet. 2013;45(10):1150-9. doi: 10.1038/ng.2742. PubMed PMID: 23974872; PubMed Central PMCID: PMCPMC3827979.

13. Bradfield JP, Taal HR, Timpson NJ, Scherag A, Lecoeur C, Warrington NM, et al. A genome-wide association meta-analysis identifies new childhood obesity loci. Nat Genet. 2012;44(5):526-31. doi: 10.1038/ng.2247. PubMed PMID: 22484627; PubMed Central PMCID: PMCPMC3370100.

14. Horikoshi M, Yaghootkar H, Mook-Kanamori DO, Sovio U, Taal HR, Hennig BJ, et al. New loci associated with birth weight identify genetic links between intrauterine growth and adult height and metabolism. Nat Genet. 2013;45(1):76-82. doi: 10.1038/ng.2477. PubMed PMID: 23202124; PubMed Central PMCID: PMCPMC3605762.

15. van der Valk RJ, Kreiner-Moller E, Kooijman MN, Guxens M, Stergiakouli E, Saaf A, et al. A novel common variant in DCST2 is associated with length in early life and height in adulthood. Hum Mol Genet. 2015;24(4):1155-68. doi: 10.1093/hmg/ddu510. PubMed PMID: 25281659; PubMed Central PMCID: PMCPMC4447786.

16. Locke AE, Kahali B, Berndt SI, Justice AE, Pers TH, Day FR, et al. Genetic studies of body mass index yield new insights for obesity biology. Nature. 2015;518(7538):197-206. doi: 10.1038/nature14177. PubMed PMID: 25673413; PubMed Central PMCID: PMCPMC4382211.

17. Schunkert H, Konig IR, Kathiresan S, Reilly MP, Assimes TL, Holm H, et al. Large-scale association analysis identifies 13 new susceptibility loci for coronary artery disease. Nat Genet. 2011;43(4):333-8. doi: 10.1038/ng.784. PubMed PMID: 21378990; PubMed Central PMCID: PMCPMC3119261.

18. Dupuis J, Langenberg C, Prokopenko I, Saxena R, Soranzo N, Jackson AU, et al. New genetic loci implicated in fasting glucose homeostasis and their impact on type 2 diabetes risk. Nat Genet. 2010;42(2):105-16. doi: 10.1038/ng.520. PubMed PMID: 20081858; PubMed Central PMCID: PMCPMC3018764.

19. Teslovich TM, Musunuru K, Smith AV, Edmondson AC, Stylianou IM, Koseki M, et al. Biological, clinical and population relevance of 95 loci for blood lipids. Nature. 2010;466(7307):707-13. doi: 10.1038/nature09270. PubMed PMID: 20686565; PubMed Central PMCID: PMCPMC3039276.

20. McCarty CA, Chisholm RL, Chute CG, Kullo IJ, Jarvik GP, Larson EB, et al. The eMERGE Network: a consortium of biorepositories linked to electronic medical records data for conducting genomic studies. BMC medical genomics. 2011;4(1):13.
